# Prevalence, sources, and predictors of soy consumption in breast cancer

**DOI:** 10.1186/1475-2891-8-2

**Published:** 2009-01-22

**Authors:** Carolyn A Lammersfeld, Jessica King, Sharon Walker, Pankaj G Vashi, James F Grutsch, Christopher G Lis, Digant Gupta

**Affiliations:** 1 Cancer Treatment Centers of America^® ^(CTCA) at Midwestern Regional Medical Center, 2610 Sheridan Road, Zion, IL, 60099, USA

## Abstract

**Background:**

A number of components in soy appear to have anticancer properties, including the isoflavones, genistein and daidzein. The use of soy by women with breast cancer is now being questioned because of the estrogen-like effects of isoflavones and possible interactions with tamoxifen. Clinicians providing nutrition counseling to these women are concerned because the availability of soy foods has increased dramatically in the past few years. The goal of this study was to quantify the intake of isoflavones in women with breast cancer.

**Methods:**

A cross-sectional study of 100 women with breast cancer treated at Cancer Treatment Centers of America^® ^between 09/03 and 02/04. Each patient completed a soy food frequency questionnaire (FFQ) that was scored by Fred Hutchinson Cancer Research Center. Demographic and clinical predictors of soy intake were evaluated using one-way non-parametric Mann Whitney test and non-parametric spearman's rank correlation.

**Results:**

Mean age was 50.5 years (std. dev. = 9.4; range 31–70) and mean BMI was 27.3 kg/m^2 ^(std. dev. = 6.75; range 17–59). Genistein and Daidzein consumption was limited to 65 patients with a mean intake of 11.6 mg/day (std. dev. = 21.9; range 0–97.4) and 7.6 mg/day (std. dev. = 14.1; range 0–68.9) respectively. Soy milk (37%) and pills containing soy, isoflavones, or "natural" estrogen (24%) were the two biggest contributors to isoflavone intake.

**Conclusion:**

Our study suggests that the isoflavone intake of breast cancer patients at our hospital was quite variable. Thirty-five patients reported no soy intake. The mean daily intake of 11.6 mg genistein and 7.4 mg daidzein, is the equivalent of less than 1/4 cup of tofu per day. This amount is higher than what has been previously reported in non-Asian American women.

## Background

Soy foods are known to have a number of health benefits, ranging from the well-documented cholesterol-lowering effects of soy protein, which may lower risk of cardiovascular disease [1], to the potential for decreasing bone loss in healthy, postmenopausal women [2,3]. A number of soy components are also thought to have anticancer properties, including isoflavones (genistein, daidzein, glycitein), phytic acid, protease inhibitors, saponins, phytosterols and lignans [4]. Indeed, in vitro research indicates that isoflavones in particular may have antiestrogenic effects [5] and rodent studies have demonstrated associations of decreased risk of mammary tumors with soy intake [6]. Furthermore, it has been suggested that the lower risk for developing breast cancer in Asian countries may be related to soy intake [7-9]. A meta-analysis by Trock, et. al found that soy intake was associated with a 14% reduced risk of breast cancer in all women [10].

However, amid this promising research, other studies have raised the question of potential risk associated with the consumption of soy by women with breast cancer due to possible estrogenic effects of soy. An animal study in ovariectomized mice by Hsieh et al. using MCF-7 estrogen receptor (ER) positive breast cancer cells showed that low (and physiologically significant) concentrations of genistein may stimulate the proliferation of these cells [11]. Another study, in ovariectomized, athymic mice implanted with MCF-7 cells, found that genistein negated the inhibitory effect of tamoxifen on MCF-7 tumor growth in vivo [12]. One of the few human studies available noted epithelial cell hyperplasia as well as increased nipple-fluid aspirate in post-menopausal women who consumed an isoflavone-rich diet [13]. Because no clear consensus about the physiological effects of soy exists, the consumption of soy foods by women with breast cancer has become a controversial issue, and the need for further research continues to exist.

Meanwhile, clinicians providing counseling to breast cancer patients are concerned because the availability, familiarity and therefore, consumption, of soy foods by the American public has increased dramatically over the past 15 years [14,15]. Numerous studies have examined the soy food intake of Asian women [16,17], Dutch women [18], US women [19] and even women at high risk for breast cancer [20], however, very limited information is available regarding the consumption of soy foods by women actually diagnosed with breast cancer. This study attempts to quantify the amount and type of soy food intake in women with breast cancer.

## Methods

### Study Population

This cross-sectional study investigated a consecutive case series of 100 new breast cancer patients treated at Cancer Treatment Centers of America^® ^(CTCA) at Midwestern Regional Medical Center (MRMC) between 09/03 and 02/04. Only patients with a histologically confirmed diagnosis of breast cancer were included in this study. Other criteria for entry into the study included a minimum age of 18 years, willingness to sign informed consent and literacy in the English language. Patients were excluded if they were unable to give informed consent or understand or cooperate with study conditions. Every new patient with a diagnosis of breast cancer that scheduled an appointment at our institution between 09/03 and 02/04 was asked to participate in this study. One hundred percent of patients consented to participate, often citing a willingness to help others with this disease, as their primary motivator.

A trained registered dietitian described the study and determined eligibility after patients signed in at the clinic. As part of the consent process, patients were assured that refusal to participate in the study would not affect their future care in any way. Patients filled in the questionnaires before undergoing any therapy at our hospital. The Institutional Review Board at MRMC approved the study.

### Questionnaire

After obtaining informed consent, each patient was asked to complete a soy food frequency questionnaire (FFQ) that was developed and validated by Fred Hutchinson Cancer Research Center. A detailed description of the FFQ has been previously published, but briefly, this 40-item soy FFQ was developed specifically to estimate soy food consumption and isoflavone intake over the past 3 months. For each food item, respondents were asked about frequency of consumption and the usual serving size consumed for each food. Options for portions size were small, medium, or large. Examples were given for a medium portion of each food item. A small portion was defined as a half of a medium portion and a large portion as one and a half times that of a medium portion. Isoflavone calculations were performed using the Nutrient Data System for Research (NDS-R) software version developed by the Nutrition Coordinating Center, University of Minnesota, Minneapolis, MN, Food and Nutrition Database. This FFQ was found to be reliable in two different studies examining two different populations [21,22]. Isoflavone values from this FFQ have also been shown to correlate well with blood levels of isoflavones [23]. In addition to completing the FFQ, subjects were also asked to detail any changes they had made to their diets since being diagnosed with cancer by answering two additional questions. The first question asked, "which response best describes your current diet?" The choices for this question included: Standard American diet, lacto-ovo vegetarian, no red meat, vegan, macrobiotic, & other with a blank for fill-in. The second question was open-ended & simply asked if any changes had been made to diet post-diagnosis. If the answer was yes, a description was requested. These responses were then categorized and coded for analysis.

### Statistical Analysis

All data were analyzed using SPSS 11.5 (SPSS Inc., Chicago, IL, USA). Means, medians and standard deviations of genistein and daidzein intake were calculated. Soy foods with highest reported consumption were determined. All continuous variables were examined for normal distribution. Demographic and clinical predictors of soy intake (genistein and daidzein intake) were evaluated using one-way non-parametric Mann Whitney test or non-parametric spearman's rank correlation depending upon the underlying distributions of the variables. Among the predictors to be evaluated were age at diagnosis, body mass index (BMI), stage at diagnosis, ethnicity, prior treatment history (newly diagnosed versus those who had failed prior treatment elsewhere), menstrual status, estrogen and progesterone receptor status, and tamoxifen use. The soy consumption was categorized into 2 groups: those who had consumed any soy food in the last three months versus those who had not. The relationship of demographic and clinical predictors was also examined with the following soy consumption variables: weekly gram weight of soy foods consumed, weekly milligrams of genistein in soy foods consumed and weekly milligrams of daidzein in soy foods consumed. The significance level for all analyses was set at alpha = 0.05 and all tests were 2-sided.

## Results

### Patient Characteristics

A total of 100 breast cancer patients were approached in the clinic between 9/03 and 2/04 and were screened for eligibility by the clinical oncology nutritionist. One Hundred patients were found to be eligible for participation. Of 100 eligible patients, 100 consented to participate and completed the soy survey. The final study sample consisted of 100 patients with a response rate of 100%. The majority of this cohort was white (81%). The mean age was 50.5 years (std. dev. = 9.4; range 31–70). The mean BMI was 27.3 (std. dev. = 6.7; range 17.1–59.2). Fifty-nine percent were ER+, 37% ER-, and 4% unknown. Twenty-nine percent were on tamoxifen, 7% on Arimidex, % 11% on both. Twenty-three percent of participants reported following a vegetarian diet. Table 1 describes the characteristics of our patient cohort in greater detail.

**Table 1:** Subject characteristics (N = 100)

**Characteristic**	**Categories**	**Number**	**Percent (%)**
Ethnicity	Caucasian	81	81
	African American	15	15
	Hispanic	3	3
	Asian	1	1

Tumor Stage at Diagnosis	Stage 0	4	4
	Stage I	21	21
	Stage II	44	44
	Stage III	17	17
	Stage IV	11	11
	Unknown	3	3

Menstrual Status	Premenopausal	53	53
	Postmenopausal	37	37
	Perimenopausal	4	4
	Unknown	6	6

ER/PR status	ER + PR +	48	48
	ER + PR -	11	11
	ER - PR +	1	1
	ER - PR -	36	36
	Unknown	4	4

Tamoxifen/Arimidex use	Neither	50	50
	Tamoxifen only	29	29
	Arimidex only	7	7
	Both	11	11
	Unknown	3	3

### Prevalence of Soy Use

The prevalence of soy consumption in our patient cohort was 65%. Consumers were defined as those who consumed at least one soy food in the past three months. The mean intake of genistein and daidzein in the previous three months was 11.6 mg/day (std. dev. = 21.9; range 0–97.4) and 7.6 mg/day (std. dev. = 14.1; range 0–68.9) respectively. Median intake of genistein and daidzein was 1 mg/day and 0.64 mg/day respectively. Table 2 provides data on the soy foods with the highest reported consumption. Table 3 provides data on the major contributors to isoflavone intake.

**Table 2:** Ten soy foods with highest reported consumption

**Soy Food**	**Percent Patients**
Soy sauce	37

Soy milk	23

Soy bars	18

Roasted soy nuts	13

Tofu	13

Veggie soy or tofu burger	12

Soy cheese	12

Packaged mixed dishes with soy or tofu	10

Tofu or soy breakfast sausage	9

Soy drinks	9

**Table 3:** Top ten contributors to isoflavone intake

**Soy Food**	**Percent Contribution**
Soymilk	37%

Pills containing soy, isoflavones, or "natural estrogens"	24%

Protein/energy bars containing soy	12%

Tofu	7%

Soy nuts	5%

Soy meat substitutes (e.g. burgers, TVP)	4%

Liquid nutrition drinks containing soy protein such as Odwalla Future Shake, Ensure Plus	3%

Soy protein powders	3%

Soy yogurt	3%

Miso soup	1%

### Predictors of Soy Use

Age at diagnosis, BMI, and weekly milligrams intake of genistein and daidzein were non-normally distributed and therefore non-parametric methods were used to analyze the relationships between them. Table 4 displays the results of Spearman Rank correlation analyses between the demographic and clinical factors and soy consumption. The results for weekly consumption of genistein and daidzein in milligrams were very similar to each other. Ethnicity and Tamoxifen use were weakly (but significantly) correlated with soy consumption. Forty-eight out of 81 Caucasians (59.3%) consumed soy whereas 15 out of 15 African-Americans (100%) consumed soy. Sixteen out of 29 (55.2%) patients with Tamoxifen use consumed soy whereas 5 out of 7 patients with Arimidex use (71.4%) consumed soy. Stage at diagnosis was significantly correlated with the three continuous soy consumption variables. The mean daily milligram of genistein consumed was 5.0, 10.8, 10.0, 0.77, and 5.1 in stages 0, I, II, III and IV respectively. The mean daily milligram of diadzein consumed was 3.2, 7.2, 6.4, 0.53, and 3.4 in stages 0, I, II, III and IV respectively. Thus, stages I and II had the highest consumption of genistein and diadzein while stage III had the lowest consumption.

**Table 4:** Spearman rank correlations between demographic and clinical factors and soy consumption (N = 100)

**Predictors**	**Soy consumption (yes versus no)**	**Weekly gram weight of soy**	**Weekly milligrams of Genistein**	**Weekly milligrams of Daidzein**
Age at Diagnosis	0.014	0.06	0.12	0.12

BMI	-0.04	-0.11	-0.11	-0.11

Ethnicity	-0.23*	0.18	0.13	0.13

Stage at Diagnosis	-0.18	-0.25*	-0.25*	-0.24*

Prior Treatment History	-0.12	-0.19	-0.18	-0.18

Menstrual Status	0.07	0.11	0.14	0.14

Estrogen/Progesterone Receptor Status	0.16	0.08	0.07	0.07

Tamoxifen Use	-0.21*	-0.20	-0.16	-0.16

* p < 0.05

An interesting relationship was observed between the change in diet after cancer diagnosis question and soy intake. The mean daily milligram of genistein consumed was 5.6 and 10.8 respectively for those who did not change their diet after cancer diagnosis (n = 30) and those who switched over to a more vegetarian diet (n = 23). Similarly, the mean daily milligram of diadzein consumed was 3.3 and 7.0 for those who did not change their diet after cancer diagnosis (n = 30) and those who switched over to a more vegetarian diet (n = 23). In the open ended question asking respondents to describe changes to their diets since diagnosis, 14 respondents mentioned soy intake. Interestingly, there were 7 responses reporting increasing soy intake since diagnosis, and 7 responses reporting decreasing soy intake since diagnosis. In addition to changing towards a vegetarian diet, other changes mentioned were decreasing sugar, increasing vegetables and fruits, decreasing portions, & decreasing fat.

## Discussion

In our study of 100 women diagnosed with breast cancer, the intake of soy isoflavones was quite variable ranging from 0 to 161.4 mg per day. This is similar to the range of 0 to > 150 mg/day reported by Frankenfeld et al. [23]. We found 35% of these subjects to be soy non-consumers, which is also similar to the findings by Frankenfeld et al. [22,23].

The mean intake of isoflavones in this population is higher than that reported in other US women and in women in the Bay Area Breast Cancer Study [19,24], but lower than that reported in Asian populations [25-27]. Isoflavone intake in the women in this study was similar to that previously reported for vegetarians [21,22]. In fact, many of the patients in this study reported making diet changes since their diagnosis. Twenty-three patients reported following a vegetarian diet.

The ten soy foods with the highest reported consumption (Table 2) contributed to 68% of isoflavone intake. Soy sauce and soy condiments had the highest reported consumption, yet contributed very little to isoflavone intake (see Table 3). Soymilk and pills containing soy, isoflavones, or "natural" estrogens contributed to 61% of total isoflavone (daidzen and genistein) intake of all participants (Table 3). An interesting finding was that these pills contributed to 24% of total isoflavone intake of all participants, yet these pills were consumed by < 9% of the participants. These pills can be very concentrated sources of isoflavones. Women consuming these pills had isoflavone intakes at the higher end of the range reported for this cohort. These women had intakes of isoflavones that exceeded 100 mg/day, which according to Messina and Wood is an intake that no more than 5% of older adults in Japan and China consume [27]. This suggests that some women with breast cancer may be consuming isoflavones in amounts above and beyond what appears to be safely consumed in Asian countries. Inquiring about isoflavone supplement intake may be one way to screen women with breast cancer for isoflavone consumption that may be excessive.

Another interesting finding was that many of the foods with high consumption were processed soy foods, including bars, burgers, cheese, packaged dishes, sausages, and drinks. In contrast, the soy consumed in Asian countries is minimally processed. A study by Allred et al. evaluated mice transplanted with MCF-7 cells that were exposed to various soy products with equal amounts of genistein aglycone equivalents. They found mice exposed to more processed soy had increased tumor growth compared to mice that received less processed soy [28]. This raises the question of whether or not we should also be concerned with the degree of processing in soy foods that are consumed by women with breast cancer. Much research still needs to be done in this area.

Several limitations of this study must be acknowledged. The patient cohort was limited to only those patients who were English-speakers and consisted largely of Caucasians with breast cancer. This study sample, therefore, is not broadly representative of breast cancer patients in general. Our hospital offers a wide range of integrative cancer treatment options including nutritional, naturopathic, mind body medicine and spiritual therapies in conjunction with conventional cancer care. It seems likely that many patients seeking care at our hospital have a particular interest in pursuing complementary therapies and may therefore have a higher prevalence of soy use than the broader population of patients with breast cancer. In fact, many of the patients in this study reported making diet changes since their diagnosis. Our sample size might not have been large enough to accommodate the number of post hoc subgroup comparisons made within this study. This study was not an attempt to scientifically evaluate the impact of soy consumption on breast cancer but rather to describe what proportion of people with breast cancer use soy foods. The non-normal distribution of age at diagnosis, BMI, and weekly milligrams intake of genistein and daidzein could be a result of relatively small sample size of our study.

FFQs have been criticized for being too vague when it comes to serving sizes. The definition of serving sizes is often open to individual interpretation. The soy FFQ used in our study defines a medium serving size for the foods in the questionnaire; and defines small and large servings in relation to the medium serving. FFQs have also been criticized for being prone to measurement error. How often one consumes a particular food is often hard to answer and/or remember. FFQs may result in underreporting based on the theory that people behave differently when observed. Finally, the number of foods on a FFQ can be limited, and preparation is not always taken into account [29]. For example, the FFQ used in this study only asked about soy foods. There are isoflavones in other foods such as beans, peas, flour, coffee, etc., which were not evaluated with this instrument. To this point, it has been reported that isoflavone containing foods other than soy make a minimal contribution to total isoflavone exposure [19]. A possible defense of this limitation is that we used a FFQ that has been validated when compared to plasma levels of isoflavones and when given at different intervals with time between measurements [21-23].

## Conclusion

Despite these limitations, this study adds to the limited information that currently exists on quantifying soy isoflavone intake in women with breast cancer. We found isoflavone intake in women with breast cancer in this study to be similar to the intake of isoflavones in vegetarians, but less than that in Asian woman. Although vegetarian diets can vary, they generally are not associated with an increase in breast cancer risk or mortality [30,31]. Potential utility of this study is that clinicians may be able to use the list of foods in Table 3 to quickly screen women with breast cancer for isoflavone intake. As was previously mentioned, asking women with breast cancer about isoflavone containing pills is one way to identify some women with intakes that may be excessive. In this cohort, soymilk, pills, and bars containing soy contributed to 73% of isoflavone intake. Asking about these three foods may be one way to quickly estimate isoflavone intake in women with breast cancer. Ideally, future studies should evaluate isoflavone intake in women with breast cancer being treated at multiple centers, in order to determine if these results are broadly representative of isoflavone intake in these women. Additional studies are also needed to determine what a safe and acceptable level of isoflavone intake is in women with breast cancer given the potential biphasic effect of isoflavone exposure.

## Competing interests

The authors declare that they have no competing interests.

## Authors' contributions

CAL was the main author of the manuscript, participated in concept, design, data collection, data analysis and data interpretation. JK and SW participated in concept, design, data collection and writing. PGV participated in concept, design and data interpretation. JFG and CGL assisted with the statistical analysis and data interpretation. DG participated in concept, design, analysis, data interpretation and writing. All authors read and approved the final manuscript.
